# Prediction on the risk population of idiosyncratic adverse reactions based on molecular docking with mutant proteins

**DOI:** 10.18632/oncotarget.21509

**Published:** 2017-10-05

**Authors:** Hongbo Xie, Diheng Zeng, Xiujie Chen, Diwei Huo, Lei Liu, Denan Zhang, Qing Jin, Kehui Ke, Ming Hu

**Affiliations:** ^1^ Department of Pharmacogenomics, College of Bioinformatics Science and Technology, Harbin Medical University, Harbin, Heilongjiang 150081, PR China; ^2^ The 2nd Affiliated Hospital of Harbin Medical University, Harbin, Heilongjiang 150081, PR China

**Keywords:** drug-induced liver injury, personalized medicine, homology-modeling, molecular simulation, risk population prediction

## Abstract

Idiosyncratic adverse drug reactions are drug reactions that occur rarely and unpredictably among the population. These reactions often occur after a drug is marketed, which means that they are strongly related to the genotype of the population. The prediction of such adverse reactions is a major challenge because of the lack of appropriate test models during the drug development process. In this study, we chose withdrawn drugs because the reasons why they were withdrawn and from which countries or regions is easily obtained. We selected Dilevalol and its chiral drug (Labetalol) as the investigatory drugs, as they have been withdrawn from a European market (Britain) because of serious hepatotoxicity. First, we searched for and obtained the Dilevalol-induced- liver-injury related protein, multidrug resistance protein 1 (MDR1), from the Comparative Toxicogenomics Database (CTD). Then, we searched and extracted 477 non-synonymous single nucleotide polymorphisms (nsSNP) on MDR1 in the dbSNP database. Second, we used the VarMod tool to predict the functional changes of MDR1 induced by these nsSNPs, from which we extracted the nsSNPs that significantly change the functions of this protein. Third, we built the three-dimensional structures of those variant proteins and used AutoDock to perform a docking study, choosing the best model to determine the sites of nsSNPs. Finally, we used the data from the 1000 Genomes Project to verify the dominant population distribution of the risk SNP. We applied the same strategy to the post-marketing drug-induced liver injury drugs to further test the feasibility of our method.

## INTRODUCTION

The liver is the major organ of drug metabolism and the main target organ of drug injury. According to statistics, at least 1100 drugs have potential hepatotoxicity. This kind of liver injury caused by drugs is known as drug-induced liver injury (DILI). DILI has different clinical manifestations, ranging from self-recovery after drug withdrawal to severe cases that lead to liver failure or even death. DILI has been a major issue for public health and a pressing issue in drug toxicology. Based on knowledge of the current drug research and development (R&D) process, DILI can lead to drug withdrawal after marketing [1] or during phases II or III of clinical trials [2]. This serious hepatotoxicity, especially DILI that is found after marketing, named idiosyncratic DILI, has a strong correlation with the genotype of the population. This kind of hepatotoxicity is unpredictable due to the lack of a genotype testing model in the drug R&D process. With the maturation of genomics and structural biology as well as the generation of high-throughput data, the data and technologies now provide a base for establishing a test model of idiosyncratic DILI. It is necessary to find effective strategies or models to predict DILI and then prevent this potential side effect.

Different Drug Response (DDR) is a common problem in clinical treatment after individual drug use. These differences range from a failure to respond to the drug to an adverse drug response (ADR) [3]. It is now clear that these individual variations are due to genomic variations from individual to individual. SNPs play an important role in the study of DDRs that are induced by genomic variations [4]. SNPs show genome wide distribution in both coding and noncoding regions. In coding regions, SNPs are classified into two types, synonymous and non-synonymous SNPs. SNPs in the coding regions that do not affect the protein sequence are known as synonymous SNPs, and SNPs that change the sequence of a protein are known as non-synonymous SNPs, which can further be divided into missense and nonsense SNPs [5, 6]. These polymorphisms on drug related genes, such as drug receptors, drug transporters and drug metabolizing enzymes, can be an important determinant of the clinical response [6].

ABC transporter superfamily genes are biologically plausible candidates for a role in DILI susceptibility, especially because some ABC transporter family gene products transport both bile acids and drugs [7]. Some genetic forms of cholestasis have been found to be associated with specific mutations in the ABCB4 (MDR3) and ABCB11 (BEP) genes [8]. When a small group of patients suffered DILI after a series of drug administrations, a report indicated that a polymorphism in the exon region of the ABCB11 was associated with cholestasis injury [9]. However, this is a very common polymorphism and has little effect on the risk of disease. No exact results show the association between ABCB11 SNPs with cholestasis liver injury. Another transporter, the ABCB1 gene, which encodes the protein MDR1, has been studied in patients with DILI due to nevirapine. In a major study of the African race, there was a significantly decreased incidence of the C3435T ABCB1 SNP [10], and a similar relationship was also reported in a smaller group of U.S. patients [11]. However, a more recent study on DILI induced by nevirapine in Europeans was unable to confirm the association of DILI with ABCB1. Even so, the sensitivity of nevirapine DILI can be affected by both HLA and ABCB1 genotypes.

We can determine what kind of gene variant may be associated with drug response, as phenotype is reflected by the functions of proteins; thus, gene mutation may lead to functional changes of proteins that cause the disease. Because of this, many protein function evaluation algorithms have been developed [12, 13]. Additionally, protein structure prediction plays an important role in the study of the relationship between proteins and diseases, but resolving the structure of a protein is a labor-intensive and time-consuming process [14]. With the further development of the genome and proteome projects, a large number of proteins with particular functions will be discovered in the future, so the requirement for protein structure prediction is increasing [15].

Predicting drug side effects is an important topic in the field of drug discovery. Several methods have been proposed to predict ADRs. Rainer Winnenburg et al. generalized enrichment analysis improves the detection of adverse drug events from biomedical literature [16]. Zhang et al. propose a novel method, the “feature selection-based multi-label k-nearest neighbor method” (FS-MLKNN), which can simultaneously determine critical feature dimensions and construct high-accuracy multi-label prediction models [17]. However, there is still room for improvements. In this study, we combined prediction on the risk population of idiosyncratic adverse reactions based on molecular docking with mutant proteins. With more and more three-dimensional structures of proteins and nucleic acids being resolved, it allows us, in many cases, to study the interactions between proteins and small molecules [18]. The molecular docking technique differs from the traditional methods that spend considerable time calculating the binding mode between biological macromolecules and substrates—this method can quickly and accurately predict the interaction between biological macromolecules and substrates [19]. Molecular docking is the process of biological macromolecules interacting with each other or various small molecules with a high specificity and affinity to form a specific complex. Compared with other methods (Genome Wide Association Study or gene expression profile), the employment of molecular docking to drug recognition can help to refine the three-dimensional structures of targets. Recently, Luo et al. identified and curated the associations between drugs and class I HLAs from the literature and demonstrated that molecular docking could differentiate the significant drug-HLA associations that lead to idiosyncratic drug reactions from the non-significant associations [20]. This indicates that molecular docking can be utilized for screening drug-HLA interactions and predicting potential idiosyncratic drug reactions. Driessche et al. presented a molecular docking analysis of the common HLA-B*57:01 variant known to be responsible for several HLA-linked adverse effects such as the abacavir hypersensitivity syndrome [21]. Our study focused on the mutation of MDR1. There have been no previous studies in this area.

## RESULTS

### Evaluation of nsSNPs on MDR1

MDR1 (Multidrug resistance protein 1) is a transmembrane protein encoded by gene ABCB1 that has a length of 1280 amino acids and effects an energy-dependent efflux pump responsible for decreased drug accumulation in multidrug-resistant cells. We input the 477 nsSNPs from the dbSNP database to VarMod and gained the protein function effect scores of the 477 nsSNPs [22, 23]. We selected all the amino acid variant sites that have scores greater than 0.7; a portion of the filter results is listed in Table 1. We set the threshold value at 0.7 because the higher the score, the greater the functional changes on protein. The nsSNV with scores less than 0.7 would have little or no effect on the functional changes of the proteins. The detailed score results are shown in the supplemental materials (Supplementary Table 1).

**Table 1:** VarMod predicted score of functional changes of amino acids

(ABCB1) Amino acid position	Damage score
R41C	0.866
Y42C	0.833
W315C	0.855
S403Y	0.829
C431W	0.866
S434I	0.803
Y490C	0.88
R543C	0.913
R547C	0.9
T558M	0.881
G579C	0.876
R580W	0.913
R588C	0.879
L772H	0.82
G894R	0.826
R958W	0.828

### MDR1 protein modeling results and docking evaluation

### MDR1 wild-type modeling results and analysis

**Figure 1:**
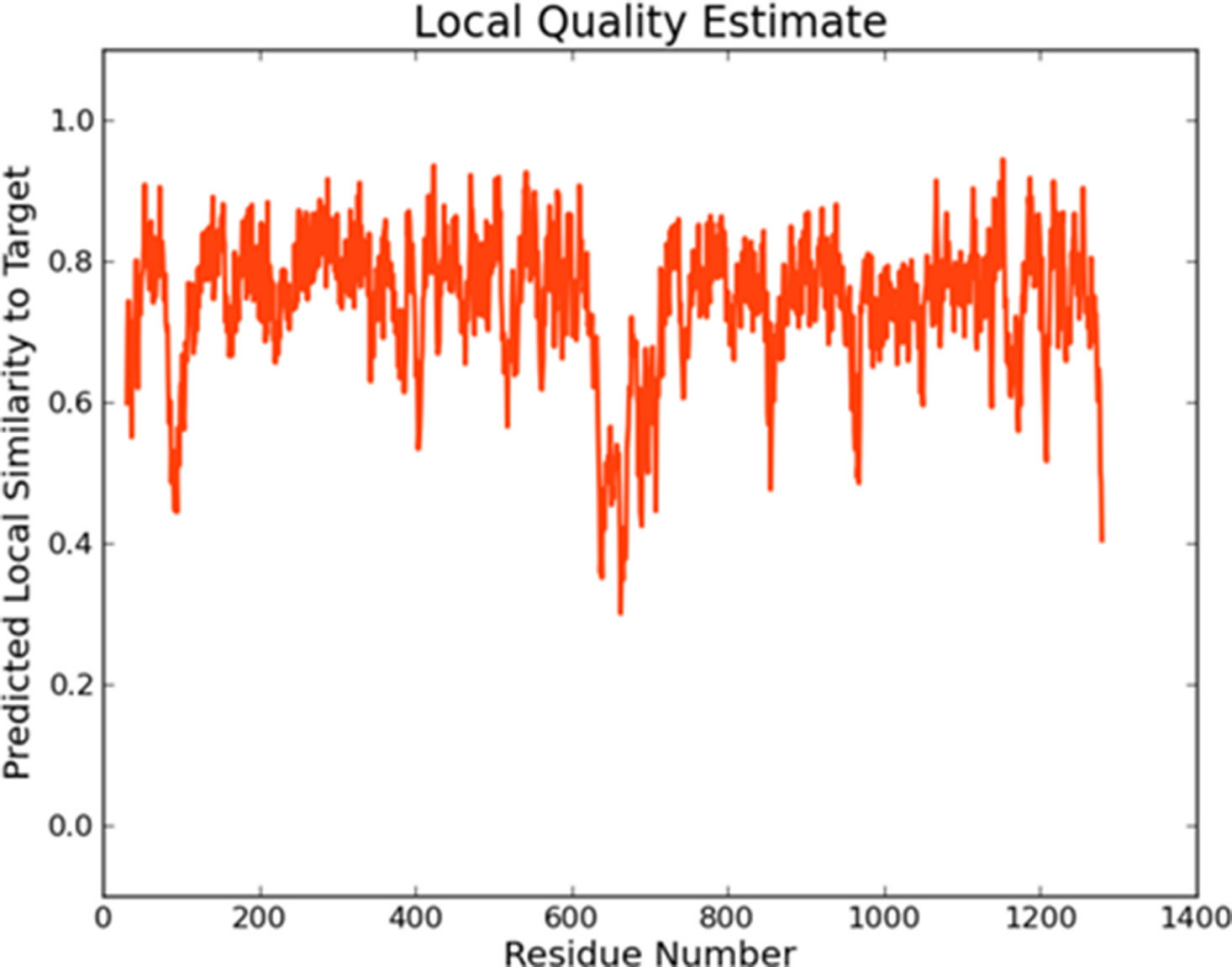
The local quality estimate of Model I

**Figure 2:**
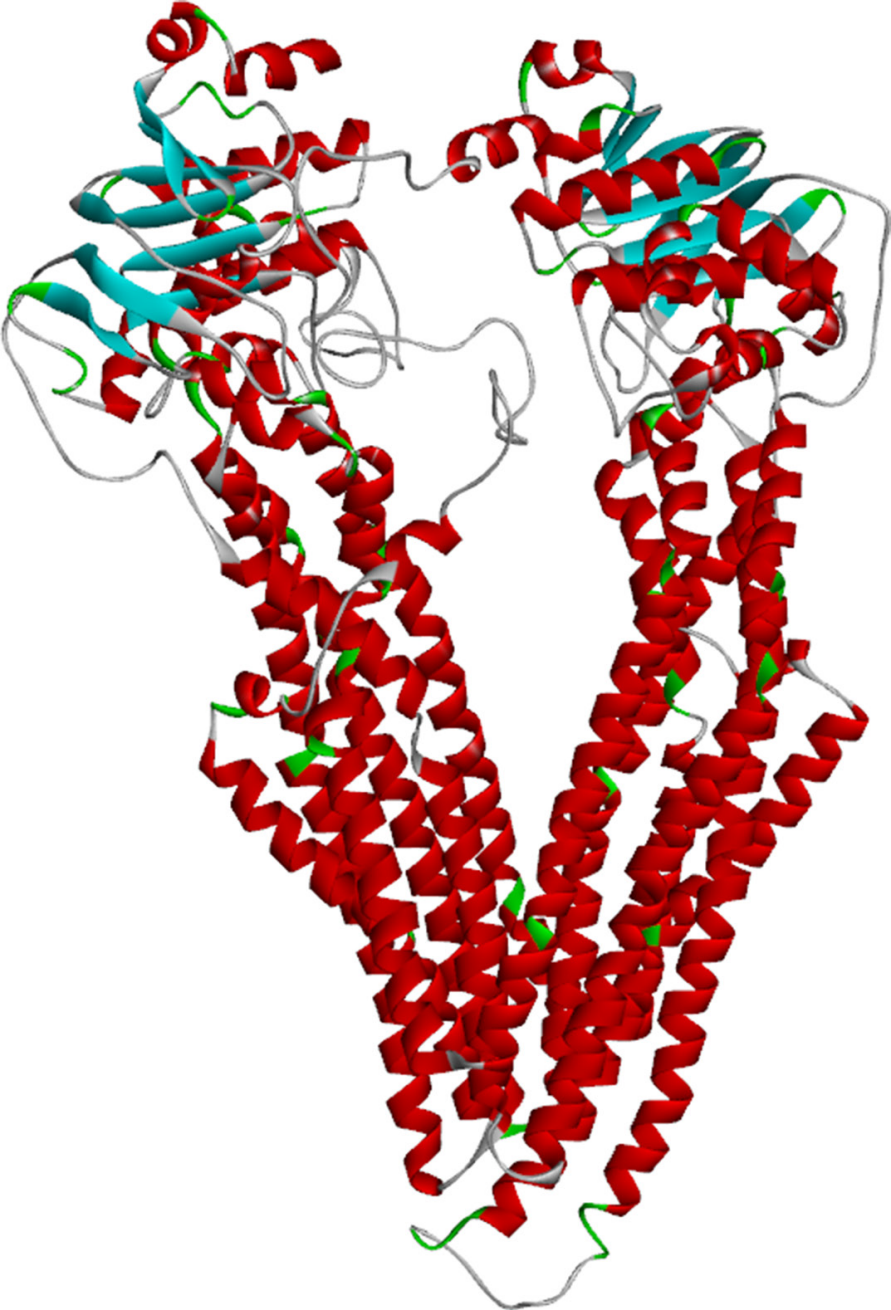
The structure of wild-type MDR1

The full length of the wild-type MDR1 protein sequence was imported into Swiss-Model [24], and 50 templates together with 3 best models were given by the tool. All three models have sequence identities greater than 87%, and the Global Model Quality Estimation (GMQE) scores were greater than 0.84. This suggested that the models were all reasonable. In the modeling result, the sequence identity of Model I was 87.26%, and the sequence coverage ranged from 31 to 1275. The local quality estimate result of Model I is shown in Figure 1. The similarities were mostly approximately 0.8, which also demonstrates the reliability of the model. The local quality estimates of the three models are shown in the supplementary information (Supplementary Figure 1). The structure of wild-type MDR1 is shown in Figure 2.

### Modeling MDR1 mutants and docking results

The binding pocket of MDR1 could not be confirmed from the PDB database, so we selected it from references. According to these references, there are two binding sites of MDR1. Binding site I consists of amino acids Ile340, Met68, Phe75, Tyr307, Phe951 and Val987 [25]. Binding site II has a center amino acid known as Arg905 [26].

The PDB files of wild-type MDR1 were imported into Discovery Studio Visualizer 4.0 to show the variant sites. After showing the region within the 6 Å around variant site Ile340, six variant sites, F303L, Y307H, Q725R, Y953C, Y953H, F983L and M986I, were obtained. All six variant types were modeled in Swiss-Model. The binding results of the seven variant types and the wild-type MDR1 with the two drugs (Labetalol and Dilevalol) are shown in Table 2.

**Table 2:** Binding scores of binding site I

Protein	Labetalol/(kcal/mol)	Dilevalol/(kcal/mol)
F303L	–6.56	–6.68
Y307H	–5.99	–6.49
Q725R	**–7.24**	**–7.49**
Y953C	–6.67	–5.39
Y953H	–5.51	–5.58
F983L	–5.93	–6.02
M986I	–6.42	–6.25
MDR1 (Wild type)	**–6.03**	**–6.48**

In the same way, five variant sites were obtained from binding site II, and the docking results are listed in Table 3.

**Table 3:** Binding scores of binding site II

Protein	Labtalol/(kcal/mol)	Dilevalol/(kcal/mol)
I160M	–3.65	–3.23
L443F	–3.21	–3.18
A900T	–3.52	–3.5
R905Q	–4.03	**–4.21**
V907I	–3.14	–3.19
MDR1 (Wild type)	–4.22	**–3.21**

Generally, the most simple and direct evaluation of docking results is the energy score. A lower energy indicates a stronger binding pattern of the ligand-receptor pair. We considered a score of less than -7 kcal/mol to represent that the ligand and receptor could bind together in the chosen site.

**Figure 3:**
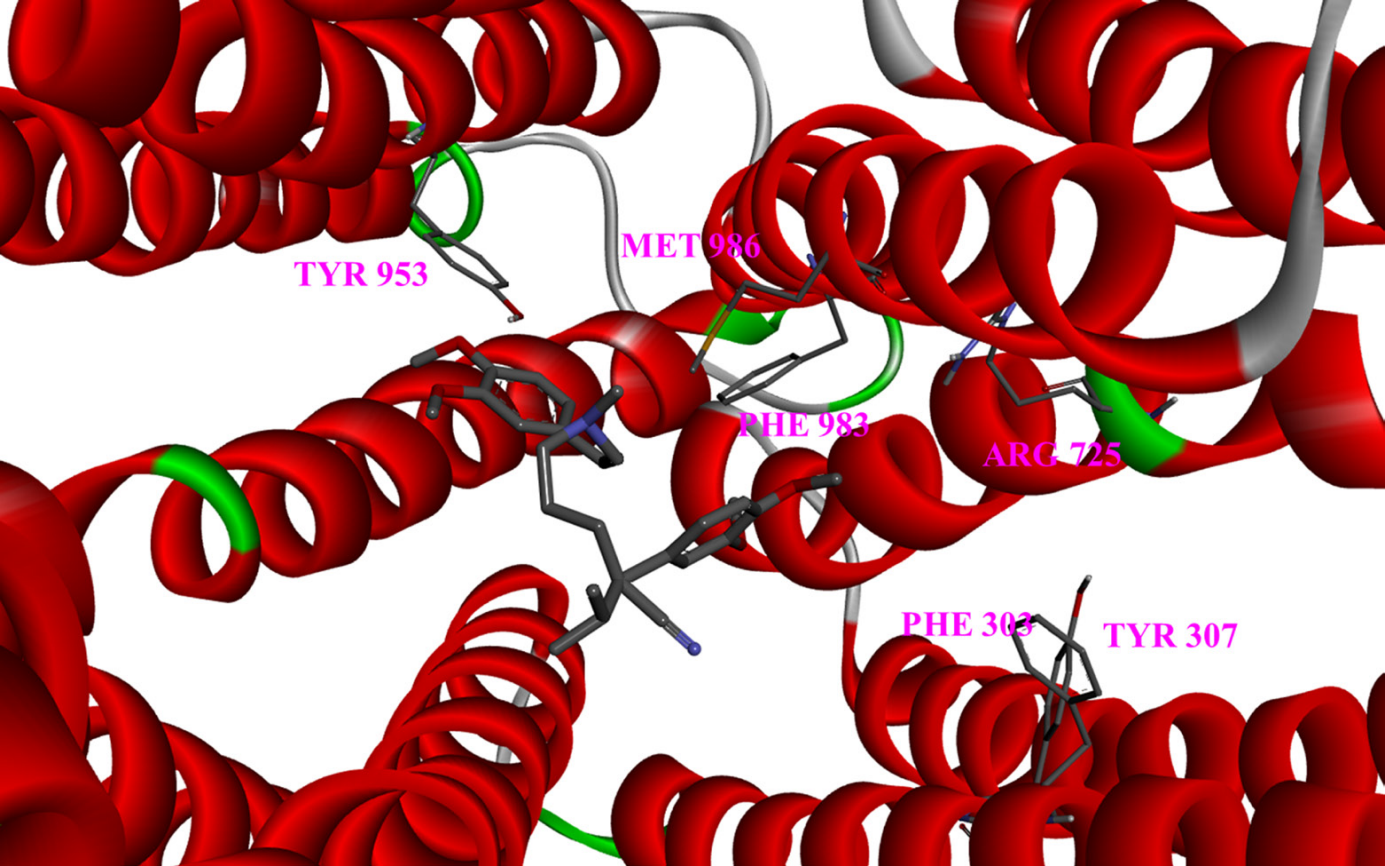
Close-up of the drug docked into the Q725R mutant Six variant sites are depicted as sticks.

By comparing the binding scores, diversity could be found between all the variant types within binding site I. Variant type Q725R (Figure 3) was the only variant that scored less than -7 kcal/mol with both drugs, lower than the wild type, indicating that Q725R could bind to both Labetalol and Dilevalol, which directly or indirectly leads to liver toxicity. Population distribution data from the 1000 Genomes Project showed that the Q725R variant only emerged in Europeans (Table 4) while other populations had no distribution of it. This result demonstrated that the Q725R variant has a great effect among the European population.

**Table 4:** Population frequency of Q725R

Population	Allele Count	Allele Number
European (Non-Finnish)	1	66674
African	0	10366
East Asian	0	8632
European (Finnish)	0	6612
Latino	0	11558
Other	0	908

### Screening of MDR1 variants induced hepatotoxicity drug

To study the mechanism of idiosyncratic DILI that is induced by the binding of MDR1 and the drug, more drugs that cause DILI by binding to the wild-type or variant MDR1 proteins were needed. First, we searched for gene ABCB1 in the GENE classification in the CTD database [27], and then selected “disease” in the results, searching DILI in the disease category, which resulted in 211 DILI-related compounds being found in the database. Then, 14 drugs that contained the word “bind” were extracted with the interaction type of chemical interaction.

In the Side Effect Resource database (SIDER), 25 post-marketing drugs were selected by DILI keyword. After overlapping with 14 DILI drugs from the CTD, five drugs were obtained. The five DILI-related drugs were Tarcrolimus, Mefloquine, Omeprazol, Verapamil, and Tamoxifen. The five drugs above were docked to MDR1 using Autodock [28]. We used binding site I and the same setting as Labetalol in Autodock. The docking results of the five DILI post-marketing drugs are shown in Table 5, and the binding conformations of Tarcrolimus, Mefloquine, Omeprazol, Verapamil and Tamoxifen for MDR1 are shown in Supplementary Figure 2. We also tested two DILI negative drugs (doxorubicin and quinidine). The results of the two drugs are provided in Supplementary Table 2 and Supplementary Table 3. The binding affinities of the two drugs were lower than the five DILI post-marketing drugs. This shows that our method is accurate.

**Table 5:** Binding scores of the five DILI post-marketing drugs

	Binding Score (kcal/mol)							
	WT	Y953H	Q725R	F303L	Y307H	Y953C	F983L	M986I
Tarcrolimus	**–6.08**	–6.1	–6.85	–6.97	**–7.07**	–6.94	–6.67	–6.29
Mefloquine	–6.34	–5.71	–6.9	–6.76	–6.28	–5.92	–6.06	–6.21
Omeprazol	–7.88	–7.25	–7.49	–7.74	–7.41	–6.95	–7.27	–7.35
Verapamil	–6.14	–5.02	–5.91	–6.11	–4.93	–5.63	–5.7	–6.08
Tamoxifen	–7.36	–6.65	–7.83	–7.94	–7.75	–7.27	–7.03	–7.04

By comparing the binding scores, diversity can be found between all the variant types. Variant Y307H was the only variant that has a score less than -7 kcal/mol when it was docking with Tarcrolimus. This indicated that it can bind better than other variants. We also found that Y307H is distrubuted in European populations (Table 6). We further searched the FDA adverse event report system and found 6 cases, all from the UK.

**Table 6:** Population frequency of the Y307H variant

Population	Allele Count	Allele Number
European (Non-Finnish)	1	66660
African	0	10368
East Asian	0	8650
European (Finnish)	0	6614
Latino	0	11564
Other	0	908

## DISCUSSION

Idiosyncratic adverse drug reaction is always related to the population genotype. Because the current drug research and development process lacks an appropriate testing model, there is a blind spot in the drug R&D process. As the affected population expands after marketing, those ADR gene carriers would suffer from serious adverse reactions that would lead to the “black box” warning on drugs or their limitation, or even their being withdrawn from the market. Thus, it is an important challenge for the drug R&D process to establish a relevant strategy to indentify the characteristics of this kind of population genotype and then predict the risk population of DILI induced by those drugs. In our research, we start by searching drugs that were withdrawn from the market due to serious DILI because the risk population is certain and its toxic sensitivity is caused by individual genetic variance, especially by nsSNPs. Literature mining is used to find the ADR-related genes, and we consider how the variance of these genes can lead to structural and functional changes on their encoded proteins and result in the cause of ADR when they interact with the drugs. Many ADRs are caused by the parent drug, the metabolized drug, or by byproducts of drug metabolism. Drug metabolism relies on the initial transport of the drug into the hepatocyte via influx transporters. The parent drug, reactive metabolites, and heavier products will then all be transported into the bile by efflux transporters as opposed to those that transport the drug into the cell. MDR1 is known as P-glycoprotein, it transports a significant amount of xenobiotics and biological compounds into the bile. While MDR1 can be found in many tissues, it also has many substrates, making it an important efflux transporter.

We explore all the mutants on the gene by performing function evaluations and then modeling the protein models according to those functional variants. Furthermore, we use drug-protein docking simulations to determine the effects of different variations on the protein after binding to the drug. We focused on idiosyncratic DILI by choosing the drugs Labetalol and Dilevalol, and our results indicated that both Q725R and Y953H have obvious docking differences from other variant types. The Q725R variant can better combine with the drug, implying that this variant can lead to changes of its protein structure by changing the unsolidified binding pattern to a closer binding, which then causes the idiosyncratic liver injury. The areal distribution of the variants is also consistent with the region that is reported to be high-DILI-occurrence, implying the effectiveness of our method in helping us discover the pattern of DILI.

To further verify the effectiveness of our method, we expanded the drugs of interest from withdrawn drugs to post-marketing drugs with functions related to MDR1-mediated DILI and performed docking simulations after constructing the variant protein. The Y307H mutant was found to be more strongly bound to Tarcrolimus. The Y307H variant is distributed only among the European population; therefore, we believe that the European population will incur a higher risk of DILI after Tarcrolimus administration.

Our method is based on the variant annotation of proteins, using a homology modeling method to build the three-dimensional protein structure and then perform molecular docking to confirm the genotype that causes serious ADR. Finally, we predicted the risk population of idiosyncratic drug toxicity by SNP distribution. This is a new attempt on idiosyncratic drug toxicity testing, and the established strategy also proved to be feasible by our results. This strategy may support a novel direction to solve the problem of the lack of an appropriate testing model for serious toxicity in the current drug R&D process.

## MATERIALS AND METHODS

### Data set

### Withdrawn drug information

The withdrawn drugs were downloaded from the DrugBank database and expanded with FDA; after literature mining [29], 173 withdrawn drugs and their associated relevant information were obtained (Supplementary Table 4), including drug names, withdrawn countries and reasons for withdrawal.

### Adverse drug reaction related protein

The Dilevalol and Labetalol related DILI data were downloaded from the CTD database. The DILI data included both chemical-gene interaction and gene-disease association. In all, 646,089 pairs of chemical-gene interaction associations were extracted together with their inference scores from the database.

### Non-synonymous SNP data set

The nsSNP data were downloaded from the dbSNP database (Build 141). We extracted all the nsSNPs on gene ABCB1 (GENE ID: 5243), and 477 nsSNPs were obtained.

### Population distribution data

All the population distribution information was downloaded from the 1000 Genomes Project, gene ABCB1 SNP of England's population. Finally, we obtained 5,497 SNP locations.

### Method

### Choosing the withdrawn drug set

We accounted for the reason for withdrawal of the withdrawn drug set. Because DILI had the highest proportion of all adverse reactions, we focused on DILI for this study. We chose the drug with a relatively simple withdrawal area because that made it easy to locate the population. Dilevalol was finally chosen because it was only withdrawn in England [30]. Dilevalol is the (R, R)-isomer of the approved drug Labetalol. It is a mixed, nonselective β blocker and selective α1 blocker. Dilevalol is the stereoisomer of Labetalol but has a similar efficacy in treating hypertension. It is reported to cause hepatotoxicity as Labetalol. Thus, the two drugs were chosen as the experimental objects.

### The extraction of the adverse drug reaction gene

The CTD database contained drug-gene interaction relationships, together with the inference scores provided by the database. The associations between the drug and gene were divided into mechanism, therapeutic or unclassified, based on the reference score. We extracted the gene that was marked as a mechanism. Only the DILI-mechanism-marked gene ABCB1 was found to be related to both Dilevalol and Labetalol. ABCB1 could bind to both drugs according to the references. The binding relationship was the premise of the docking process below.

### The extraction of non-synonymous single nucleotide polymorphisms

Gene IDs were inputted to the dbSNP database to obtain all the SNP variations on the gene. All the missense-marked SNPs were extracted. The reference SNP (rs), number of dbSNP, amino acid positions and amino acid substitutions were also extracted.

### Effects of single nucleotide polymorphisms on protein function

VarMod is an online tool to predict the impact of non-synonymous SNPs on proteins. It uses the ligand binding sites and protein-protein interactions to do the modeling and combines the characteristics of residue conservation with other features to identify the functional nsSNVs. This online tool finally uses machine learning methods (SVM) to provide a global prediction that combines different independent analysis results. We extracted all the non-synonymous SNPs in ABCB1, containing 477 different variants. Then, we used VarMod to predict the influence of the 477 SNP sites on the protein function. We selected scores greater than 0.7 for the variance sites, defined as functional non-synonymous SNPs.

### Screening of mutant proteins and construction of the three-dimensional structure model of the protein

As there are a great number of nsSNPs, we selected amino acids that were within 6 Å of the drug binding site. Swiss-Model is a fully automatic, externally free online protein structure homology modeling tool. We submitted the ABCB1 protein sequence from the UniProt database and used the website in AUTOMATED mode. The Swiss-Model server automates building the homology model by first searching for a suitable template for constructing a reference-based model. Following this, the model was subjected to strained angle correction, and quality control parameters were estimated [31]. The target sequence homology model needed a follow-up test to view its rationality for further use. There are many templates of the homology modeling structures, such as 4Q9J, 4M2S and 4KSC.

### Docking and binding score

To explore the key residues of the Dilevalol binding site of the MDR1, molecular docking of Labetalol and dilevalol to the MDR1 was performed using AutoDock 4.2. The model of MDR1 was converted to PDBQT format using AutoDock Tools (ADT), version 1.5.6 (http://mgltools.scripps.edu). Then, Kollman united atom partial charges were assigned for the receptor. The grid size for the search space was set at 60 Å × 60 Å × 60 Å, centered on the binding pocket of MDR1, with a default grid point spacing of 0.375 Å. The Lamarckian genetic algorithm was used with a population size of 10 dockings and energy evaluations. Those results were clustered according to the root-mean-square deviation (RMSD) criterion.

## SUPPLEMENTARY MATERIALS FIGURES AND TABLES






