# Supplementary material for: Prediction on the risk population of idiosyncratic adverse reactions based on molecular docking with mutant proteins
Source: Oncotarget. 2017 Oct 5;8(56):95568–76. doi: 10.18632/oncotarget.21509 (PMC5707043; doi:10.18632/oncotarget.21509)
Supplement: Supplementary file 1 [file oncotarget-08-95568-s001.pdf]

Prediction on the risk population of idiosyncratic adverse reactions based on molecular docking with mutant proteins

SUPPLEMENTARY MATERIALS

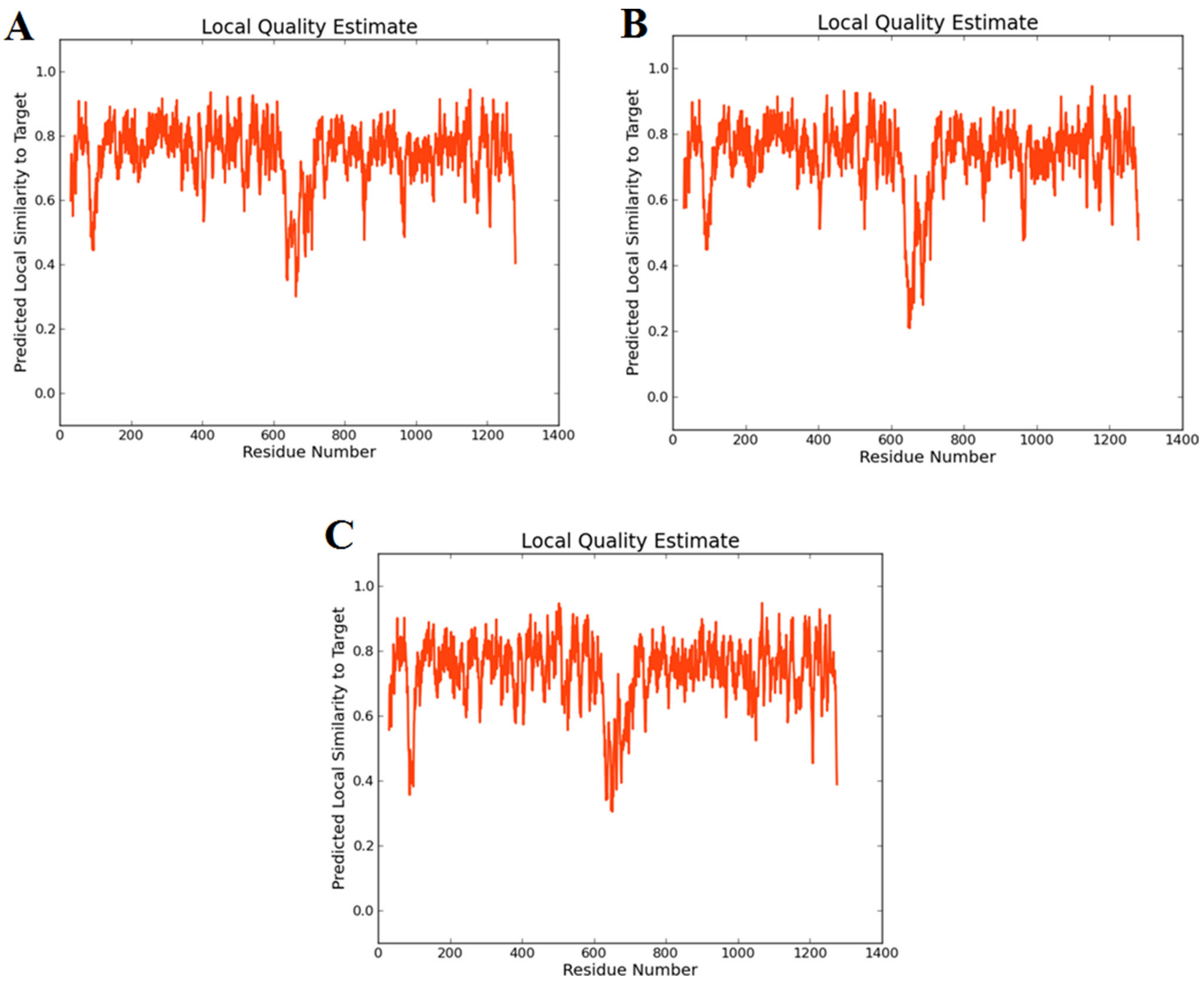

Supplementary Figure 1: The local quality estimate of Model I (**A**), Model II (**B**) and Model III (**C**).

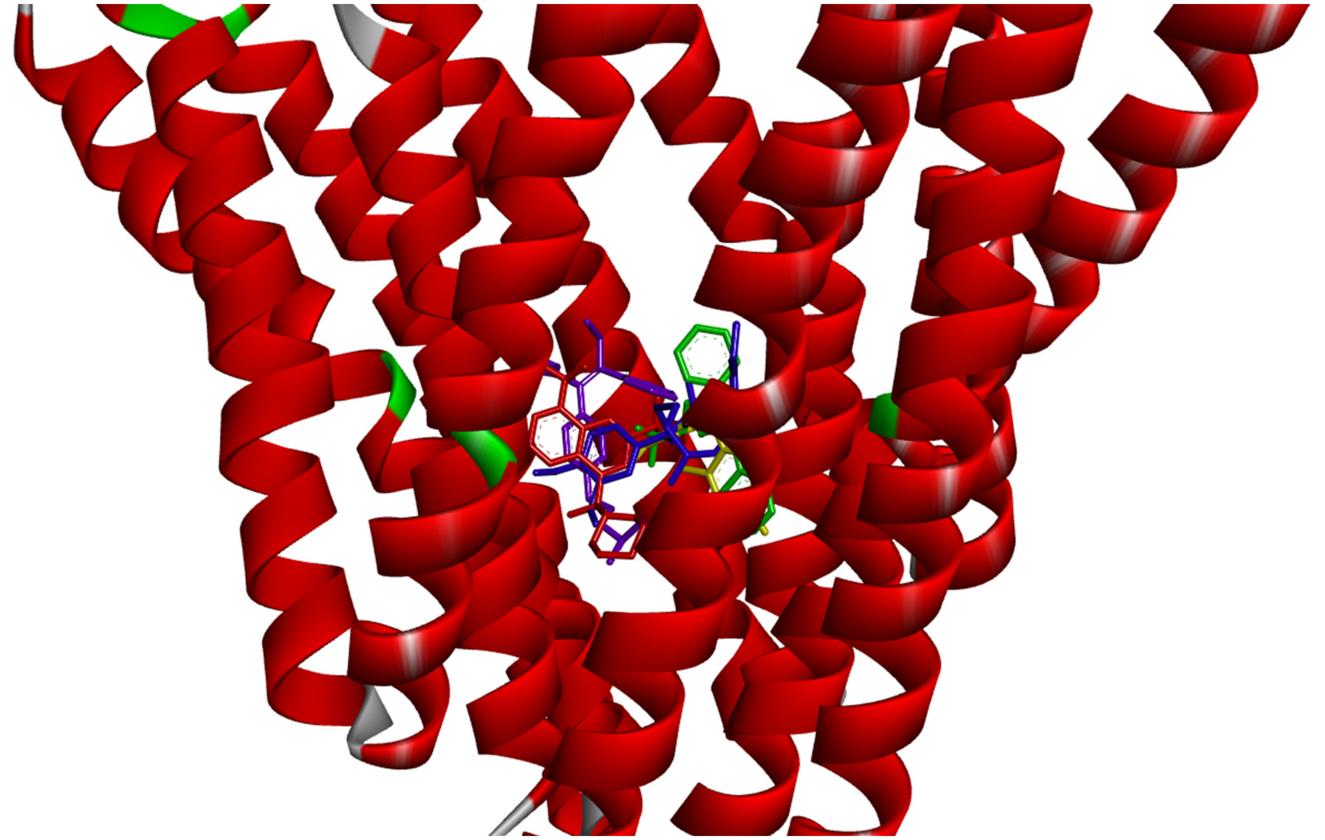

Supplementary Figure 2: The binding conformations of Tarcrolimus, Mefloquine, Omeprazol, Verapamil and Tamoxifen for MDR1.

Supplementary Table 1: VarMod predicted score of functional changes of amino acid. See Supplementary_Table_1.

Supplementary Table 2: Binding score of binding site I

Protein	Doxorubicin/(kcal/mol)	Quinidine/(kcal/mol)
F303L	–5.32	–6.25
Y307H	–5.60	–6.67
Q725R	–5.17	–5.69
Y953C	–4.12	–5.92
Y953H	–5.32	–4.34
F983L	–5.25	–5.67
M986I	–5.91	–7.13
MDR1 (Wild type)	–5.81	–4.53

Supplementary Table 3: Binding score of binding site II

Protein	Doxorubicin/(kcal/mol)	Quinidine/(kcal/mol)
I160M	–3.31	–3.48
L443F	–3.11	–3.05
A900T	–2.98	–3.23
R905Q	–3.83	–3.79
V907I	–3.01	–2.89
MDR1 (Wild)	–3.51	–2.78

Supplementary Table 4: The information of 173 withdrawn drugs. See Supplementary_Table_4.
