# Supplementary material for: Prediction on the risk population of idiosyncratic adverse reactions based on molecular docking with mutant proteins
Source: Oncotarget. 2017 Oct 5;8(56):95568–76. doi: 10.18632/oncotarget.21509 (PMC5707043; doi:10.18632/oncotarget.21509)
Supplement: Supplementary file 2 [file oncotarget-08-95568-s002.doc]

**Supplementary Table 1: VarMod predicted score of functional changes of amino acid**

| Varant | VarMod Probability | Binding site | Conservation |
| --- | --- | --- | --- |
| D2G | 0.17 | - | 0 |
| G5V | 0.23 | - | 0 |
| D6H | 0.3 | - | 0 |
| R7G | 0.07 | - | 0 |
| N8K | 0.07 | - | 0 |
| K12Q | 0.06 | - | 0 |
| F17S | 0.11 | - | 0 |
| F17L | 0.06 | - | 0 |
| K18Q | 0.18 | - | 0 |
| L19V | 0.16 | 1 | 0 |
| L19Q | 0.28 | 1 | 0 |
| N21D | 0.15 | 1 | 0 |
| D26V | 0.25 | 1 | 0.23 |
| K28R | 0.22 | 1 | 0.15 |
| P32T | 0.33 | 1 | 0.22 |
| V34I | 0.24 | 1 | 0.45 |
| M39I | 0.45 | 1 | 0.72 |
| M39V | 0.3 | 1 | 0.72 |
| R41C | 0.87 | 1 | 0.73 |
| R41H | 0.75 | 1 | 0.73 |
| Y42C | 0.83 | 1 | 0.59 |
| S43A | 0.34 | 1 | 0.51 |
| N44S | 0.25 | 1 | 0.32 |
| N44D | 0.25 | 1 | 0.32 |
| K48N | 0.57 | 1 | 0.35 |
| L49F | 0.29 | 1 | 0.5 |
| L49S | 0.53 | 1 | 0.5 |
| Y50C | 0.36 | 1 | 0.66 |
| M51T | 0.56 | 1 | 0.32 |
| V53G | 0.55 | 1 | 0.65 |
| I60L | 0.25 | 1 | 0.56 |
| A63T | 0.31 | 1 | 0.43 |
| T76I | 0.46 | 1 | 0.55 |
| I78T | 0.4 | 1 | 0.7 |
| A80E | 0.41 | 1 | 0.13 |
| N84H | 0.45 | 1 | 0 |
| M89T | 0.2 | 1 | 0 |
| T93I | 0.12 | 1 | 0 |
| N94K | 0.18 | 1 | 0 |
| D97Y | 0.13 | 1 | 0 |
| N99S | 0.18 | 1 | 0 |
| D100G | 0.2 | 1 | 0 |
| G102R | 0.27 | 1 | 0 |
| G102V | 0.13 | 1 | 0 |
| F103S | 0.17 | 1 | 0.08 |
| F104L | 0.12 | 1 | 0.1 |
| M105L | 0.22 | 1 | 0.23 |
| E109K | 0.24 | 1 | 0.28 |
| D110N | 0.3 | 1 | 0.51 |
| D110G | 0.3 | 1 | 0.51 |
| M111V | 0.23 | 1 | 0.29 |
| Y114F | 0.55 | 1 | 0.43 |
| G120E | 0.64 | 1 | 0.55 |
| A123V | 0.44 | 1 | 0.39 |
| V127G | 0.47 | 1 | 0.34 |
| Y130H | 0.61 | 1 | 0.37 |
| G141V | 0.78 | 1 | 0.72 |
| R142K | 0.37 | 1 | 0.7 |
| I144T | 0.31 | 1 | 0.25 |
| H145Y | 0.35 | 1 | 0.56 |
| F151I | 0.63 | 1 | 0.7 |
| F152V | 0.52 | 1 | 0.44 |
| I155V | 0.34 | 1 | 0.68 |
| M156L | 0.31 | 1 | 0.51 |
| M156I | 0.27 | 1 | 0.51 |
| R157Q | 0.45 | 1 | 0.79 |
| I160M | 0.35 | 1 | 0.47 |
| F163V | 0.57 | 1 | 0.83 |
| V165G | 0.45 | 1 | 0.36 |
| H166Y | 0.51 | 1 | 0.32 |
| D167N | 0.32 | 1 | 0.42 |
| V168I | 0.32 | 1 | 0.6 |
| V168A | 0.27 | 1 | 0.6 |
| T173P | 0.73 | 1 | 0.7 |
| R174Q | 0.66 | 1 | 0.56 |
| L175F | 0.69 | 1 | 0.51 |
| V179A | 0.46 | 1 | 0.3 |
| K181M | 0.79 | 1 | 0.62 |
| N183S | 0.4 | 1 | 0.62 |
| G187S | 0.75 | 1 | 0.7 |
| D188V | 0.76 | 1 | 0.77 |
| D188E | 0.44 | 1 | 0.77 |
| F193L | 0.25 | 1 | 0.5 |
| M197V | 0.33 | 1 | 0.52 |
| A198P | 0.65 | 1 | 0.54 |
| T199A | 0.45 | 1 | 0.67 |
| F200L | 0.25 | 1 | 0.41 |
| I205V | 0.28 | 1 | 0.56 |
| R210C | 0.76 | 1 | 0.5 |
| K213E | 0.37 | 1 | 0.73 |
| A220T | 0.35 | 1 | 0.38 |
| I221M | 0.55 | 1 | 0.42 |
| S222G | 0.47 | 1 | 0.74 |
| A230S | 0.3 | 1 | 0.3 |
| A230G | 0.26 | 1 | 0.3 |
| V231L | 0.2 | 1 | 0.34 |
| S237P | 0.63 | 1 | 0.32 |
| D241G | 0.45 | 1 | 0.42 |
| L244F | 0.55 | 1 | 0.21 |
| A246V | 0.66 | 1 | 0.8 |
| A250P | 0.77 | 1 | 0.68 |
| I261V | 0.32 | 0.16 | 0.79 |
| R262K | 0.25 | 0.08 | 0.76 |
| I265T | 0.38 | 0.16 | 0.65 |
| A266V | 0.41 | 0.19 | 0.79 |
| K272T | 0.56 | 0.58 | 0.58 |
| N278S | 0.31 | 0.79 | 0.31 |
| N280H | 0.36 | 0.94 | 0.69 |
| K285E | 0.34 | 1 | 0.31 |
| I287V | 0.32 | 1 | 0.59 |
| A292V | 0.37 | 1 | 0.39 |
| T294P | 0.61 | 1 | 0.29 |
| N296S | 0.34 | 1 | 0.39 |
| S298F | 0.43 | 1 | 0.36 |
| I299V | 0.37 | 1 | 0.7 |
| G300D | 0.6 | 1 | 0.26 |
| F303L | 0.41 | 1 | 0.45 |
| Y307F | 0.3 | 1 | 0.34 |
| Y307H | 0.61 | 1 | 0.34 |
| A308V | 0.55 | 1 | 0.5 |
| W315C | 0.86 | 1 | 0.74 |
| T319N | 0.68 | 1 | 0.63 |
| L320V | 0.48 | 1 | 0.56 |
| V321I | 0.23 | 1 | 0.32 |
| L322F | 0.63 | 1 | 0.35 |
| G329R | 0.71 | 1 | 0.28 |
| L332R | 0.72 | 1 | 0.51 |
| V338L | 0.44 | 1 | 0.56 |
| V345A | 0.37 | 1 | 0.76 |
| A348T | 0.34 | 1 | 0.41 |
| I352T | 0.53 | 1 | 0.41 |
| A354T | 0.66 | 1 | 0.69 |
| A356T | 0.56 | 1 | 0.27 |
| G360A | 0.64 | 1 | 0.69 |
| A361V | 0.62 | 1 | 0.67 |
| F366L | 0.32 | 1 | 0.35 |
| I369V | 0.37 | 1 | 0.58 |
| N371K | 0.32 | 1 | 0.35 |
| N371Y | 0.69 | 1 | 0.35 |
| P373A | 0.49 | 1 | 0.18 |
| S374N | 0.41 | 1 | 0.7 |
| S377G | 0.55 | 1 | 0.42 |
| S379L | 0.62 | 1 | 0.33 |
| S381R | 0.31 | 1 | 0.77 |
| H383N | 0.51 | 1 | 0.44 |
| N387D | 0.5 | 1 | 0.49 |
| I388S | 0.6 | 1 | 0.37 |
| E393A | 0.39 | 1 | 0.66 |
| E393K | 0.62 | 1 | 0.66 |
| V397I | 0.28 | 1 | 0.34 |
| H398L | 0.63 | 1 | 0.83 |
| S400N | 0.52 | 1 | 0.88 |
| S403Y | 0.83 | 1 | 0.85 |
| R404Q | 0.51 | 1 | 0.53 |
| E406K | 0.53 | 1 | 0.55 |
| V407G | 0.6 | 1 | 0.43 |
| V407F | 0.62 | 1 | 0.43 |
| K416N | 0.31 | 1 | 0.58 |
| K416R | 0.29 | 1 | 0.58 |
| T422M | 0.53 | 1 | 0.54 |
| C431W | 0.87 | 1 | 0.79 |
| K433E | 0.74 | 1 | 0.8 |
| S434I | 0.8 | 1 | 0.79 |
| L439P | 0.78 | 1 | 0.54 |
| M440V | 0.37 | 1 | 0.72 |
| M440I | 0.42 | 1 | 0.72 |
| L443F | 0.45 | 1 | 0.88 |
| E448K | 0.67 | 1 | 0.78 |
| M450T | 0.27 | 1 | 0.64 |
| D454E | 0.56 | 1 | 0.61 |
| G455E | 0.7 | 1 | 0.42 |
| D457G | 0.68 | 1 | 0.63 |
| I461T | 0.62 | 1 | 0.7 |
| N462K | 0.69 | 1 | 0.53 |
| V463L | 0.44 | 1 | 0.47 |
| R464M | 0.78 | 1 | 0.63 |
| R464G | 0.57 | 1 | 0.63 |
| R464S | 0.37 | 1 | 0.63 |
| R467Q | 0.67 | 1 | 0.41 |
| V473M | 0.74 | 1 | 0.56 |
| P477T | 0.68 | 0.84 | 0.48 |
| T482I | 0.75 | 0.88 | 0.74 |
| R489C | 0.56 | 1 | 0.67 |
| R489H | 0.78 | 1 | 0.67 |
| Y490C | 0.88 | 1 | 0.76 |
| R492C | 0.42 | 1 | 0.41 |
| R492H | 0.7 | 1 | 0.41 |
| V495I | 0.57 | 1 | 0.58 |
| M497V | 0.37 | 1 | 0.47 |
| D498G | 0.53 | 1 | 0.57 |
| I500T | 0.65 | 1 | 0.41 |
| V504L | 0.5 | 1 | 0.55 |
| E506K | 0.49 | 1 | 0.73 |
| A507G | 0.7 | 1 | 0.86 |
| N508S | 0.71 | 1 | 0.76 |
| D511G | 0.62 | 1 | 0.86 |
| M514V | 0.35 | 1 | 0.34 |
| D521G | 0.61 | 1 | 0.82 |
| T522S | 0.55 | 0.93 | 0.45 |
| E526D | 0.21 | 0.64 | 0.6 |
| A529P | 0.73 | 0.66 | 0.76 |
| S532G | 0.61 | 0.58 | 0.79 |
| K536R | 0.69 | 0.74 | 0.89 |
| K536Q | 0.79 | 0.74 | 0.89 |
| R538S | 0.74 | 0.85 | 0.72 |
| A540T | 0.79 | 0.97 | 0.8 |
| R543C | 0.91 | 1 | 0.79 |
| A544T | 0.72 | 1 | 0.6 |
| R547H | 0.55 | 1 | 0.72 |
| R547C | 0.9 | 1 | 0.72 |
| D555G | 0.73 | 1 | 0.76 |
| A557G | 0.67 | 0.87 | 0.81 |
| T558M | 0.88 | 0.81 | 0.84 |
| L561F | 0.75 | 0.52 | 0.85 |
| E566K | 0.54 | 0.63 | 0.39 |
| V571M | 0.76 | 0.93 | 0.74 |
| D574E | 0.37 | 1 | 0.57 |
| D574N | 0.42 | 1 | 0.57 |
| D574V | 0.71 | 1 | 0.57 |
| G579C | 0.88 | 1 | 0.78 |
| R580Q | 0.51 | 1 | 0.83 |
| R580W | 0.91 | 1 | 0.83 |
| T581S | 0.62 | 1 | 0.72 |
| A586T | 0.77 | 1 | 0.91 |
| R588C | 0.88 | 0.85 | 0.75 |
| T591A | 0.64 | 0.93 | 0.7 |
| R593C | 0.34 | 1 | 0.51 |
| R593H | 0.67 | 1 | 0.51 |
| A595T | 0.54 | 1 | 0.72 |
| D596N | 0.29 | 1 | 0.29 |
| V597I | 0.41 | 1 | 0.78 |
| A599T | 0.6 | 1 | 0.48 |
| G600D | 0.7 | 1 | 0.53 |
| F601I | 0.34 | 1 | 0.36 |
| D602N | 0.49 | 1 | 0.33 |
| D603E | 0.55 | 1 | 0.79 |
| D603H | 0.57 | 1 | 0.79 |
| I606F | 0.65 | 1 | 0.46 |
| V607M | 0.78 | 1 | 0.83 |
| D613Y | 0.66 | 1 | 0.63 |
| E614D | 0.56 | 1 | 0.75 |
| G620D | 0.64 | 1 | 0.38 |
| F623L | 0.41 | 1 | 0.33 |
| K624R | 0.34 | 1 | 0.73 |
| N633D | 0.23 | 1 | 0.11 |
| V635A | 0.16 | 1 | 0.17 |
| A641G | 0.17 | 1 | 0.12 |
| D642V | 0.11 | 1 | 0.17 |
| S646I | 0.29 | 1 | 0.13 |
| E647K | 0.11 | 1 | 0 |
| I648T | 0.11 | 1 | 0.1 |
| A650G | 0.11 | 1 | 0.13 |
| N656S | 0.16 | 1 | 0.23 |
| D657G | 0.11 | 1 | 0.1 |
| S661N | 0.14 | 1 | 0.12 |
| L662R | 0.34 | 1 | 0.14 |
| K665Q | 0.21 | 1 | 0.14 |
| R666I | 0.39 | 1 | 0.23 |
| T668A | 0.16 | 1 | 0.16 |
| R669C | 0.19 | 1 | 0.15 |
| R669H | 0.13 | 1 | 0.15 |
| R670K | 0.11 | 1 | 0.14 |
| S671N | 0.19 | 1 | 0.07 |
| S671R | 0.38 | 1 | 0.07 |
| R673C | 0.17 | 1 | 0 |
| R673H | 0.11 | 1 | 0 |
| D679E | 0.11 | 1 | 0.16 |
| R680G | 0.14 | 1 | 0.22 |
| I692V | 0.21 | 0.43 | 0.33 |
| P694L | 0.24 | 0.63 | 0.37 |
| M701V | 0.33 | 1 | 0.42 |
| T706N | 0.46 | 1 | 0.76 |
| V713G | 0.65 | 1 | 0.72 |
| G714A | 0.48 | 1 | 0.35 |
| Q725R | 0.68 | 1 | 0.82 |
| A727V | 0.43 | 1 | 0.68 |
| I736M | 0.62 | 1 | 0.27 |
| I736K | 0.63 | 1 | 0.27 |
| I736L | 0.22 | 1 | 0.27 |
| T740I | 0.43 | 1 | 0.19 |
| D744E | 0.18 | 1 | 0.19 |
| R749Q | 0.19 | 1 | 0.37 |
| N753D | 0.24 | 1 | 0.29 |
| F759V | 0.53 | 1 | 0.46 |
| I765V | 0.23 | 1 | 0.4 |
| I768V | 0.21 | 1 | 0.41 |
| L772H | 0.82 | 1 | 0.63 |
| G774R | 0.72 | 1 | 0.43 |
| T776K | 0.72 | 1 | 0.73 |
| R789Q | 0.3 | 0.71 | 0.23 |
| M791T | 0.32 | 0.63 | 0.41 |
| M791V | 0.27 | 0.63 | 0.41 |
| R794Q | 0.45 | 0.44 | 0.45 |
| S795T | 0.44 | 0.4 | 0.61 |
| S795C | 0.77 | 0.4 | 0.61 |
| L797I | 0.48 | 0.25 | 0.51 |
| D800E | 0.3 | 0.09 | 0.55 |
| D800N | 0.64 | 0.09 | 0.55 |
| V801M | 0.2 | 0.01 | 0.46 |
| N809S | 0.53 | 0.19 | 0.51 |
| G812R | 0.75 | 0.4 | 0.45 |
| A813G | 0.54 | 0.38 | 0.65 |
| A819T | 0.59 | 0.66 | 0.38 |
| I829V | 0.3 | 1 | 0.63 |
| G830V | 0.68 | 1 | 0.33 |
| I836V | 0.22 | 1 | 0.39 |
| I836N | 0.64 | 1 | 0.39 |
| T837A | 0.36 | 1 | 0.55 |
| N839S | 0.47 | 1 | 0.35 |
| G846E | 0.69 | 1 | 0.46 |
| I849M | 0.45 | 1 | 0.61 |
| Y853C | 0.78 | 1 | 0.45 |
| Y853N | 0.68 | 1 | 0.45 |
| L860F | 0.38 | 1 | 0.42 |
| I864T | 0.52 | 1 | 0.39 |
| I864V | 0.2 | 1 | 0.39 |
| P866S | 0.64 | 1 | 0.51 |
| V874A | 0.42 | 1 | 0.55 |
| A883T | 0.56 | 1 | 0.2 |
| L884P | 0.45 | 1 | 0.4 |
| G894R | 0.83 | 1 | 0.56 |
| I896M | 0.74 | 1 | 0.62 |
| A897T | 0.4 | 1 | 0.42 |
| T898A | 0.46 | 1 | 0.71 |
| A900T | 0.7 | 1 | 0.51 |
| R905Q | 0.74 | 1 | 0.83 |
| V907I | 0.39 | 1 | 0.53 |
| E913K | 0.61 | 1 | 0.33 |
| K915N | 0.66 | 1 | 0.61 |
| H918Y | 0.25 | 1 | 0.29 |
| M919K | 0.7 | 1 | 0.77 |
| S923N | 0.39 | 1 | 0.65 |
| Q925K | 0.32 | 1 | 0.18 |
| V926I | 0.32 | 1 | 0.6 |
| Y928S | 0.68 | 1 | 0.46 |
| L932S | 0.52 | 1 | 0.5 |
| I937L | 0.27 | 1 | 0.38 |
| T945I | 0.65 | 1 | 0.6 |
| A947S | 0.52 | 1 | 0.41 |
| M948T | 0.41 | 1 | 0.39 |
| Y953C | 0.73 | 1 | 0.64 |
| Y953H | 0.42 | 1 | 0.64 |
| C956Y | 0.76 | 1 | 0.71 |
| R958W | 0.83 | 1 | 0.56 |
| R958Q | 0.37 | 1 | 0.56 |
| L963F | 0.73 | 1 | 0.64 |
| H966N | 0.28 | 1 | 0.39 |
| E972K | 0.21 | 1 | 0.25 |
| D973A | 0.5 | 1 | 0.6 |
| D973N | 0.35 | 1 | 0.6 |
| V974I | 0.5 | 1 | 0.58 |
| F978I | 0.37 | 1 | 0.49 |
| A980P | 0.69 | 1 | 0.53 |
| F983L | 0.33 | 1 | 0.53 |
| M986I | 0.28 | 1 | 0.46 |
| V988M | 0.37 | 1 | 0.62 |
| G989V | 0.68 | 1 | 0.49 |
| S992N | 0.52 | 1 | 0.52 |
| Y998S | 0.62 | 1 | 0.39 |
| A999T | 0.44 | 0.97 | 0.64 |
| A1001G | 0.58 | 0.91 | 0.53 |
| I1009T | 0.45 | 0.6 | 0.25 |
| M1010V | 0.24 | 0.46 | 0.59 |
| E1013D | 0.26 | 0.51 | 0.5 |
| T1015N | 0.46 | 0.32 | 0.72 |
| D1019N | 0.53 | 0.47 | 0.41 |
| T1023M | 0.61 | 0.68 | 0.33 |
| G1025D | 0.59 | 0.8 | 0.27 |
| M1027L | 0.25 | 0.83 | 0.43 |
| M1027K | 0.44 | 0.83 | 0.43 |
| P1028L | 0.23 | 0.92 | 0.34 |
| N1029S | 0.17 | 0.89 | 0.22 |
| T1030A | 0.33 | 0.79 | 0.36 |
| L1031S | 0.24 | 0.73 | 0.34 |
| E1032G | 0.3 | 0.71 | 0.74 |
| F1037L | 0.2 | 0.32 | 0.32 |
| G1038D | 0.28 | 0.32 | 0.42 |
| V1040A | 0.36 | 0.26 | 0.27 |
| V1041I | 0.36 | 0.18 | 0.85 |
| Y1044F | 0.63 | 0 | 0.84 |
| T1046P | 0.79 | 0 | 0.85 |
| P1048S | 0.27 | 0.05 | 0.54 |
| P1048L | 0.49 | 0.05 | 0.54 |
| I1050L | 0.22 | 0.12 | 0.42 |
| P1051A | 0.33 | 0.01 | 0.7 |
| G1055R | 0.73 | 0.2 | 0.65 |
| G1055E | 0.66 | 0.2 | 0.65 |
| S1057R | 0.52 | 0.3 | 0.52 |
| G1063A | 0.6 | 0.61 | 0.66 |
| G1063C | 0.88 | 0.61 | 0.66 |
| T1065M | 0.78 | 0.45 | 0.53 |
| A1067V | 0.63 | 0.25 | 0.69 |
| A1067T | 0.67 | 0.25 | 0.69 |
| L1068V | 0.56 | 0.12 | 0.79 |
| S1072R | 0.75 | 0 | 0.79 |
| C1074W | 0.87 | 0 | 0.79 |
| K1076E | 0.77 | 0 | 0.84 |
| T1078P | 0.72 | 0 | 0.46 |
| Q1081R | 0.59 | 0.02 | 0.75 |
| L1083P | 0.77 | 0.19 | 0.74 |
| L1083V | 0.54 | 0.19 | 0.74 |
| R1085Q | 0.68 | 0.25 | 0.77 |
| R1085W | 0.89 | 0.25 | 0.77 |
| D1088N | 0.39 | 0.15 | 0.71 |
| K1093E | 0.28 | 0.45 | 0.62 |
| V1094M | 0.57 | 0.35 | 0.31 |
| K1099Q | 0.2 | 0.62 | 0.57 |
| V1106I | 0.24 | 0.42 | 0.42 |
| Q1107P | 0.66 | 0.54 | 0.58 |
| Q1107E | 0.31 | 0.54 | 0.58 |
| W1108R | 0.78 | 0.63 | 0.61 |
| L1109I | 0.62 | 0.49 | 0.85 |
| R1110Q | 0.59 | 0.38 | 0.43 |
| G1114S | 0.66 | 0.37 | 0.61 |
| I1115T | 0.67 | 0.28 | 0.79 |
| P1120H | 0.83 | 0.21 | 0.5 |
| I1121N | 0.78 | 0.35 | 0.77 |
| L1122V | 0.64 | 0.48 | 0.82 |
| C1125G | 0.7 | 0.8 | 0.74 |
| I1127V | 0.34 | 0.77 | 0.37 |
| E1129K | 0.5 | 0.81 | 0.88 |
| N1130I | 0.85 | 0.71 | 0.78 |
| D1135E | 0.47 | 0.64 | 0.42 |
| N1136S | 0.44 | 0.68 | 0.32 |
| S1137I | 0.56 | 0.83 | 0.37 |
| R1138Q | 0.24 | 0.87 | 0.19 |
| R1138W | 0.3 | 0.87 | 0.19 |
| S1141T | 0.27 | 0.97 | 0.39 |
| E1144K | 0.64 | 0.92 | 0.64 |
| V1146M | 0.29 | 0.83 | 0.31 |
| A1149V | 0.58 | 0.66 | 0.52 |
| A1152S | 0.66 | 0.55 | 0.77 |
| I1154M | 0.79 | 0.56 | 0.8 |
| A1156P | 0.34 | 0.71 | 0.86 |
| A1156G | 0.45 | 0.71 | 0.86 |
| I1158M | 0.68 | 0.78 | 0.37 |
| E1159K | 0.33 | 0.87 | 0.41 |
| P1162T | 0.31 | 0.99 | 0.42 |
| K1168E | 0.56 | 0.89 | 0.64 |
| V1169I | 0.56 | 0.77 | 0.78 |
| R1183S | 0.66 | 0.34 | 0.62 |
| R1183H | 0.74 | 0.34 | 0.62 |
| A1185D | 0.76 | 0.38 | 0.78 |
| R1188H | 0.8 | 0.5 | 0.77 |
| R1188G | 0.75 | 0.5 | 0.77 |
| Q1193R | 0.31 | 0.62 | 0.81 |
| H1195Y | 0.56 | 0.57 | 0.66 |
| T1203M | 0.84 | 0.08 | 0.83 |
| A1205T | 0.57 | 0.22 | 0.75 |
| E1209K | 0.57 | 0.5 | 0.81 |
| E1211A | 0.55 | 0.31 | 0.65 |
| E1216K | 0.62 | 0.43 | 0.78 |
| A1221P | 0.68 | 0.4 | 0.54 |
| E1223D | 0.41 | 0.5 | 0.6 |
| R1225H | 0.79 | 0.44 | 0.83 |
| I1228V | 0.22 | 0.2 | 0.55 |
| I1230V | 0.39 | 0 | 0.76 |
| R1233H | 0.42 | 0.17 | 0.77 |
| R1233C | 0.87 | 0.17 | 0.77 |
| T1236I | 0.7 | 0.26 | 0.73 |
| I1237V | 0.26 | 0.22 | 0.6 |
| A1240S | 0.62 | 0.41 | 0.74 |
| D1241E | 0.36 | 0.46 | 0.31 |
| D1241N | 0.26 | 0.46 | 0.31 |
| V1244A | 0.28 | 0.24 | 0.71 |
| G1249D | 0.69 | 0.1 | 0.36 |
| V1251I | 0.21 | 0.26 | 0.42 |
| K1252R | 0.25 | 0.37 | 0.8 |
| T1256K | 0.78 | 0.6 | 0.91 |
| H1257Y | 0.57 | 0.55 | 0.45 |
| M1270T | 0.43 | 0.16 | 0.49 |
| T1277A | 0.13 | 1 | 0.23 |
| R1279C | 0.46 | - | 0 |
| R1279H | 0.09 | - | 0 |
| Q1280P | 0.19 | - | - |
