# Supplementary material for: Prediction on the risk population of idiosyncratic adverse reactions based on molecular docking with mutant proteins
Source: Oncotarget. 2017 Oct 5;8(56):95568–76. doi: 10.18632/oncotarget.21509 (PMC5707043; doi:10.18632/oncotarget.21509)
Supplement: Supplementary file 3 [file oncotarget-08-95568-s003.doc]

**Supplementary Table 4: The information of 173 withdrawn drugs**

| Drug | Formula | DrugBank ID |
| --- | --- | --- |
| L-Tryptophan | C11H12N2O2 | DB00150 |
| Ethchlorvynol | C7H9ClO | DB00189 |
| Troglitazone | C24H27NO5S | DB00197 |
| Chlorotrianisene | C23H21ClO3 | DB00269 |
| Ethoxzolamide | C9H10N2O3S2 | DB00311 |
| Tolcapone | C14H11NO5 | DB00323 |
| Terfenadine | C32H41NO2 | DB00342 |
| Grepafloxacin | C19H22FN3O3 | DB00365 |
| Thiethylperazine | C22H29N3S2 | DB00372 |
| Dydrogesterone | C21H28O2 | DB00378 |
| Dexrazoxane | C11H16N4O4 | DB00380 |
| Tacrine | C13H14N2 | DB00382 |
| Phenylpropanolamine | C9H13NO | DB00397 |
| Remoxipride | C16H23BrN2O3 | DB00409 |
| Acetohexamide | C15H20N2O4S | DB00414 |
| Metocurine Iodide | C40H48I2N2O6 | DB00416 |
| Lindane | C6H6Cl6 | DB00431 |
| Cerivastatin | C26H34FNO5 | DB00439 |
| Loracarbef | C16H16ClN3O4 | DB00447 |
| Metharbital | C9H14N2O3 | DB00463 |
| Hexylcaine | C16H23NO2 | DB00473 |
| Tridihexethyl | C21H36NO | DB00505 |
| Bentiromide | C23H20N2O5 | DB00522 |
| Mephenytoin | C12H14N2O2 | DB00532 |
| Rofecoxib | C17H14O4S | DB00533 |
| Chlormerodrin | C5H11ClHgN2O2 | DB00534 |
| Fenfluramine | C12H16F3N | DB00574 |
| Valdecoxib | C16H14N2O3S | DB00580 |
| Cisapride | C23H29ClFN3O4 | DB00604 |
| Astemizole | C28H31FN4O | DB00637 |
| Dextropropoxyphene | C22H29NO2 | DB00647 |
| Sulfametopyrazine | C11H12N4O3S | DB00664 |
| Isoflurophate | C6H14FO3P | DB00677 |
| Thioridazine | C21H26N2S2 | DB00679 |
| Moricizine | C22H25N3O4S | DB00680 |
| Trovafloxacin | C20H15F3N4O3 | DB00685 |
| Diphemanil Methylsulfate | C21H27NO4S | DB00729 |
| Hetacillin | C19H23N3O4S | DB00739 |
| Zileuton | C11H12N2O2S | DB00744 |
| Hexachlorophene | C13H6Cl6O2 | DB00756 |
| Potassium Chloride | ClK | DB00761 |
| Benzquinamide | C22H32N2O5 | DB00767 |
| Roxithromycin | C41H76N2O15 | DB00778 |
| Haloprogin | C9H4Cl3IO | DB00793 |
| Halazepam | C17H12ClF3N2O | DB00801 |
| Carprofen | C15H12ClNO2 | DB00821 |
| Cinoxacin | C12H10N2O5 | DB00827 |
| Levamisole | C11H12N2S | DB00848 |
| Chlorphenesin | C9H11ClO3 | DB00856 |
| Alprenolol | C15H23NO2 | DB00866 |
| Suprofen | C14H12O3S | DB00870 |
| Flupentixol | C23H25F3N2OS | DB00875 |
| Bitolterol | C28H31NO5 | DB00901 |
| Phenformin | C10H15N5 | DB00914 |
| Etretinate | C23H30O3 | DB00926 |
| Alosetron | C17H18N4O | DB00969 |
| Ethinamate | C9H13NO2 | DB01031 |
| Carphenazine | C23H29N3O2S | DB01038 |
| Thalidomide | C13H10N2O4 | DB01041 |
| Tegaserod | C16H23N5O | DB01079 |
| Sibutramine | C17H26ClN | DB01105 |
| Methyprylon | C10H17NO2 | DB01107 |
| Trilostane | C20H27NO3 | DB01108 |
| Cefadroxil | C16H17N3O5S | DB01140 |
| Nefazodone | C25H32ClN5O2 | DB01149 |
| Amdinocillin | C15H23N3O3S | DB01163 |
| Chlormezanone | C11H12ClNO3S | DB01178 |
| Pergolide | C19H26N2S | DB01186 |
| Dexfenfluramine | C12H16F3N | DB01191 |
| Flecainide | C17H20F6N2O3 | DB01195 |
| Encainide | C22H28N2O2 | DB01228 |
| Pemoline | C9H8N2O2 | DB01230 |
| Diphenidol | C21H27NO | DB01231 |
| Chlorprothixene | C18H18ClNS | DB01239 |
| Bepridil | C24H34N2O | DB01244 |
| Mibefradil | C29H38FN3O3 | DB01388 |
| Temafloxacin | C21H18F3N3O3 | DB01405 |
| Aminophenazone | C13H17N3O | DB01424 |
| Bezitramide | C31H32N4O2 | DB01459 |
| Fencamfamine | C15H21N | DB01463 |
| Ethylestrenol | C20H32O | DB01493 |
| Chloral betaine | C7H14Cl3NO4 | DB01494 |
| Etryptamine | C12H16N2 | DB01546 |
| Fenproporex | C12H16N2 | DB01550 |
| Chlorphentermine | C10H14ClN | DB01556 |
| Tolrestat | C16H14F3NO3S | DB02383 |
| Oxyphenbutazone | C19H20N2O3 | DB03585 |
| Phenacetin | C10H13NO2 | DB03783 |
| Nimesulide | C13H12N2O5S | DB04743 |
| Benoxaprofen | C16H12ClNO3 | DB04812 |
| Bithionol | C12H6Cl4O2S | DB04813 |
| Bunamiodyl | C15H16I3NO3 | DB04814 |
| Clioquinol | C9H5ClINO | DB04815 |
| Danthron | C14H8O4 | DB04816 |
| Metamizole | C13H16N3NaO4S | DB04817 |
| Iproniazid | C9H13N3O | DB04818 |
| Methapyrilene | C14H20ClN3S | DB04819 |
| Nialamide | C16H18N4O2 | DB04820 |
| Nomifensine | C16H18N2 | DB04821 |
| Oxeladin | C20H33NO3 | DB04822 |
| Oxyphenisatin | C20H15NO3 | DB04823 |
| Phenolphthalein | C20H14O4 | DB04824 |
| Prenylamine | C24H27N | DB04825 |
| Thenalidine | C17H22N2S | DB04826 |
| Ethyl carbamate | C3H7NO2 | DB04827 |
| Zomepirac | C15H14ClNO3 | DB04828 |
| Lysergic Acid Diethylamide | C20H25N3O | DB04829 |
| Buformin | C6H15N5 | DB04830 |
| Ticrynafen | C13H8Cl2O4S | DB04831 |
| Zimelidine | C16H17BrN2 | DB04832 |
| Methaqualone | C16H14N2O | DB04833 |
| Rapacuronium | C37H61N2O4 | DB04834 |
| Amineptine | C22H28ClNO2 | DB04836 |
| Clofedanol | C17H20ClNO | DB04837 |
| Ximelagatran | C24H35N5O5 | DB04898 |
| Telaprevir | C36H53N7O6 | DB05521 |
| Sertindole | C24H26ClFN4O | DB06144 |
| Eflornithine | C6H12F2N2O2 | DB06243 |
| Sitaxentan | C18H15ClN2O6S2 | DB06268 |
| Halcinonide | C24H32ClFO5 | DB06786 |
| Mangafodipir | C22H30MnN4O14P2 | DB06796 |
| Nonoxynol-9 | C33H60O10 | DB06804 |
| Plicamycin | C52H76O24 | DB06810 |
| Azapropazone | C16H18N4O2 | DB07402 |
| Hexestrol | C18H22O2 | DB07931 |
| Ethyl biscoumacetate | C22H16O8 | DB08794 |
| Aminopterin | C19H20N8O5 | DB08878 |
| Formestane | C19H26O3 | DB08905 |
| Magaldrate | AlHMgO5S | DB08938 |
| Isoxsuprine | C18H23NO3 | DB08941 |
| Isoxicam | C14H13N3O5S | DB08942 |
| Isoaminile | C16H24N2 | DB08944 |
| Iopanoic acid | C11H12I3NO2 | DB08946 |
| Iodamide | C12H11I3N2O4 | DB08948 |
| Inositol nicotinate | C42H30N6O12 | DB08949 |
| Indoramin | C22H25N3O | DB08950 |
| Indoprofen | C17H15NO3 | DB08951 |
| Indenolol | C15H21NO2 | DB08952 |
| Indalpine | C15H20N2 | DB08953 |
| Ifenprodil | C21H27NO2 | DB08954 |
| Ibuproxam | C13H19NO2 | DB08955 |
| Hydroxydione | C21H32O3 | DB08956 |
| Hexoprenaline | C22H32N2O6 | DB08957 |
| Glibornuride | C18H26N2O4S | DB08962 |
| Gemeprost | C23H38O5 | DB08964 |
| Fusafungine | C33H57N3O9 | DB08965 |
| Fominoben | C21H24ClN3O3 | DB08968 |
| Flurothyl | C4H4F6O | DB08969 |
| Fluprednidene | C22H27FO5 | DB08970 |
| Fluocortolone | C22H29FO4 | DB08971 |
| Flumequine | C14H12FNO3 | DB08972 |
| Fluclorolone acetonide | C24H29Cl2FO5 | DB08973 |
| Flubendazole | C16H12FN3O3 | DB08974 |
| Florantyrone | C20H14O3 | DB08975 |
| Floctafenine | C20H17F3N2O4 | DB08976 |
| Fentonium | C31H34NO4 | DB08978 |
| Fendiline | C23H25N | DB08980 |
| Etilefrine | C10H15NO2 | DB08985 |
